# A four-domain approach of frailty explored in the Doetinchem Cohort Study

**DOI:** 10.1186/s12877-017-0595-0

**Published:** 2017-08-30

**Authors:** Sandra H van Oostrom, Daphne L van der A, M Liset Rietman, H Susan J Picavet, Manon Lette, W M Monique Verschuren, Simone R de Bruin, Annemieke M W Spijkerman

**Affiliations:** 1 Centre for Nutrition, Prevention and Health Services, National Institute of Public Health and the Environment, P.O. Box 1, 3720 Bilthoven, BA The Netherlands; 2 Julius Center for Health Sciences and Primary Care, University Medical Center Utrecht, Utrecht, the Netherlands

**Keywords:** Frailty, Multidimensional, Lifestyle, Physical activity, Sleep, Prospective cohort

## Abstract

**Background:**

Accumulation of problems in physical, psychological, cognitive, or social functioning is characteristic for frail individuals. Using a four-domain approach of frailty, this study explored how sociodemographic and lifestyle factors, life events and health are associated with frailty.

**Methods:**

The study sample included 4019 men and women (aged 40–81 years) examined during the fifth round (2008–2012) of the Doetinchem Cohort Study. Four domains of frailty were considered: physical (≥4 of 8 criteria: unintentional weight loss, exhaustion, strength, perceived health, walking, balance, hearing and vision impairments), psychological (2 criteria: depressive symptoms, mental health), cognitive (<10th percentile on global cognitive functioning), and social frailty (≥2 of 3 criteria: loneliness, social support, social participation). Logistic regression was used to study the cross-sectional association of sociodemographic factors, lifestyle, life events and chronic diseases with frailty domains.

**Results:**

About 17% of the population was frail on one or more domains. Overlap between the frailty domains was limited since 82% of the frail population was frail on one domain only. Low educated respondents were at higher risk of being psychologically and socially frail. Having multiple diseases was associated with a higher risk of being physically and psychologically frail. Being physically active was consistently associated with a lower risk of frailty on each of the four domains. Short or long sleep duration was associated with a higher risk of being physically, psychologically, and socially frail.

**Conclusions:**

Sociodemographic factors, lifestyle and multimorbidity contributed differently to the four frailty domains. It is important to consider multiple frailty domains since this helps to identify different groups of frail people, and as such to provide tailored care and support. Lifestyle factors including physical activity, smoking and sleep duration were associated with multiple domains of frailty.

**Electronic supplementary material:**

The online version of this article doi: 10.1186/s12877-017-0595-0) contains supplementary material, which is available to authorized users.

## Background

With ageing, changes occur in physical, psychological, cognitive, and social functioning. Accumulation of problems in one or more of these domains of functioning is characteristic for frail people. Originally, frailty was mainly focused on the physical problems that older people encounter, such as in Fried’s popular ‘phenotype of frailty’ [1]. Broader definitions of frailty, looking beyond physical functioning, have now been put forward [2–4] one of which is the definition by Gobbens et al. [5]. According to them, frailty is ‘a dynamic state affecting an individual who experiences losses in one or more domains of human functioning (physical, psychological, social) caused by the influence of a range of variables and which increases the risk of adverse outcomes’. A multidimensional approach to frailty is coherent with the interdisciplinary diagnostic process used in the Comprehensive Geriatric Assessment for frail older people, which also examines physical, mental (including both psychological and cognitive functioning), and social functioning [6, 7].

Frailty often leads to restrictions in mobility and reduced self-reliance, and a greater risk of clinically significant adverse outcomes such as hospitalization, institutionalization and mortality [1, 4, 8–10]. In several European countries it is government policy to stimulate older people to participate in society and to live at home for as long as possible [11, 12]. Primary prevention of frailty is therefore needed, directed at both delaying the onset of frailty and slowing down the frailty process as prevention of frailty might eventually lead to prevention or postponement of hospitalization and institutionalization of elderly people. Insight in factors that are associated with the presence of frailty is a first step to assist the identification of potentially vulnerable groups. For the physically frail, a series of socio-demographic, lifestyle, and health-related factors have been shown to be associated with frailty, such as age, female sex, cardiovascular diseases, multimorbidity, BMI, and smoking [13]. As part of a broad frailty definition, little is known about factors associated with the psychological and social domains of frailty. Especially, the association between lifestyle factors and frailty has rarely been studied [13].

Recently, the concept of cognitive frailty has been proposed [14]. Since there is increasing support for the idea of cognitive frailty being a separate frailty domain [15, 16], a four-domain approach of frailty was adopted for the current study including the physical, cognitive, psychological and social domains of frailty. It was recently shown that the overlap between these frailty domains was limited, which implicates that the domains largely entail distinct populations and frailty prevention may target multiple frailty domains [17].

In this study, we explored how sociodemographic factors, lifestyle factors, life events, biological risk factors and chronic diseases were associated with physical, psychological, cognitive, and social frailty in a population-based study of men and women aged 40–81 years.

## Methods

### Study population

Data of men and women aged 40–81 years participating in the Doetinchem Cohort Study were used for the current study. The Doetinchem Cohort Study is an ongoing population-based cohort study aimed to study the impact of (changes in) lifestyle and biological risk factors on various aspects of health and wellbeing of men and women, aged 20–59 years at baseline, from the Netherlands. A total of 12,405 participants (response rate 62%) were first examined in 1987–1991 (round 1). Of those, a random sample of 7768 participants was re-invited to be examined in 1993–1997 (round 2, *n* = 6113), 1998–2002 (round 3, *n* = 4916), 2003–2007 (round 4, *n* = 4520) and 2008–2012 (round 5, *n* = 4019). The response rates for all follow-up measurements varied between 75% and 80%. For the current study, we used data from the fifth examination round. Written informed consent was obtained from all participants. The Medical Ethics Committees of the Netherlands Organization of Applied Scientific Research and the University of Utrecht approved the study. Full details of the study have been reported elsewhere [18].

### Conceptual model of frailty

**Fig. 1:**
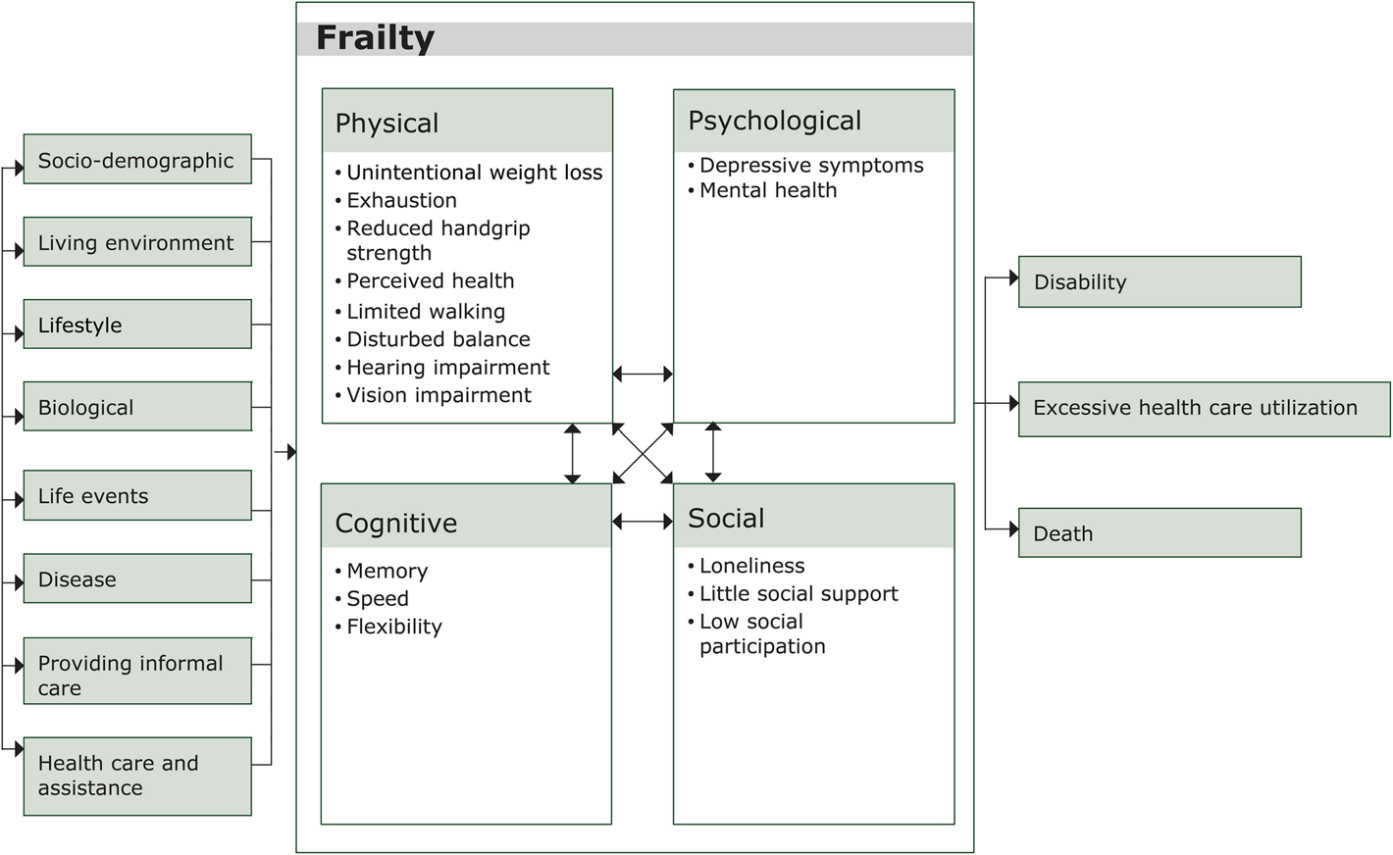
Adapted version of the integral conceptual model of frailty, based on Gobbens [19]

The integral conceptual model of frailty which includes the physical, psychological, and social domains of frailty was the basis of our study [19]. In the original model, the psychological domain included feelings of anxiety and depression, a decline in coping, and a decline in cognitive functioning. For the current study, the conceptual model was extended with a fourth domain, being cognitive frailty (Fig. 1) [14]. The rationale for adding a fourth domain was that limitations in functioning due to anxious and depressive feelings (i.e. the psychological domain) are considered to be fundamentally different from limitations in functioning due to e.g. memory problems (i.e. cognitive functioning). In addition, we noticed that cognitive functioning was previously not consistently positioned in one domain of frailty; it belonged either to the physical or the psychological domain [1, 7, 19]. Furthermore, we added several potential determinants of frailty to the model, including health care and support, and providing informal care (Fig. 1). Inadequate formal care or support for chronic diseases or acute disorders may lead to a strong deterioration of physical, cognitive, psychological functioning and increase the risk to become frail. Informal caregiving is also related to negative health outcomes [20].

### Operationalization of frailty domains

The frailty criteria per domain were based on the Tilburg Frailty Indicator (TFI, Table 1) [21]. The original TFI was not included in our questionnaires. For each item on the TFI we identified the best possible proxy in our questionnaires and data-collection. A detailed description of the criteria can be found in Additional file 1. Participants were considered to be physically frail if they fulfilled ≥4 of 8 frailty criteria described by Gobbens [21, 22]. Participants were considered to be cognitively frail when scoring <10th percentile on a global cognitive functioning score based on memory, speed, and flexibility. Cognitive scores were adjusted for level of education and number of tests performed during follow-up. Psychological frailty was defined as fulfilling both criteria for depression [23] and for poor mental health [24]. Social frailty was defined as meeting ≥2 of 3 criteria using the Loneliness scale [25], Social Support List-12 [26] and a questionnaire about social participation from the Dutch Municipal Health Services Elderly Monitor [27]. Overall frailty was defined as all participants being frail on one or more domains.

**Table 1:** Overview of the criteria used to operationalize physical, cognitive, psychological, and social frailty

Domains	Criteria	Cut-off	Based on
Physical frailty	- Unintentional weight loss - Exhaustion - Low handgrip strength - Perceived health - Limited in walking - Disturbed balance - Hearing impairment - Vision impairment	≥4 criteria	- Unintentional weight loss: >5% weight loss between round 4 and 5 and not being on a diet - Exhaustion: 2 questions of the Center for Epidemiologic Studies Depression scale (CES-D) [23] - Handgrip strength: dynamometer, sex-specific cut-off stratified for BMI [1] - Perceived health: one question of 36-Item Short-Form Health Survey (SF-36) [24, 62] - Self-reported 100 m walking - Tandem Stand Balance Test - 3 questions regarding hearing - 3 questions regarding vision
Cognitive frailty	- Low global cognitive functioning	<10th percentile	Global cognitive functioning score based on tests for memory, speed and flexibility [63]: 15 Words Verbal Learning Test Stroop Colour–Word Test Word Fluency Test Letter Digit Substitution Test
Psychological frailty	- Depressive symptoms - Mental health	=2 criteria	- Center for Epidemiologic Studies Depression scale (CES-D) [23] - Mental Health Inventory 5 (MHI-5) [24, 64]
Social frailty	- Loneliness - Low social support - Limited social participation	≥2 criteria	- Loneliness Scale [25, 65] - Social Support List-12 (SSL-12) [26] - Questionnaire Dutch Elderly Monitor [27]

### Other measurements

#### Socio-demographic factors

Level of education was categorized into low (intermediate secondary education or less), intermediate (intermediate vocational and higher secondary education) and high (higher vocational education or university). Work status was defined as having a paid job (including salaried employment and self-employed) or being unemployed. Household composition was defined as living alone or not living alone (living with a partner, with children, with parents or other adults). Being married also included registered partnership.

#### Lifestyle

For defining smoking status, we distinguished current smokers and non-smokers. To establish whether or not people had a healthy diet, the World Health Organisation’s dietary recommendations for the prevention of chronic disease were applied [28]. Score on the healthy diet indicator ranged from 0 to 9 and was based on the sum of the number of nutrients (out of a group of seven nutrients) and the number of products from two food groups for which intake was within the recommended range [29]. Being physically active was defined as adherence to the Dutch physical activity guideline, which recommends 30 min of moderate to vigorous physical activity per day on at least 5 days per week [30]. The average sleep duration per 24-h period was assessed in four categories: 5 h or less, 6 h, 7 or 8 h, and 9 h or more. Alcohol consumption was assessed in four categories: never, not anymore, <1 glass a week, and ≥1 glass a week [31].

#### Life events

We determined recent life events (i.e. widowhood, divorce) by evaluating potential changes in marital status between round 4 and round 5. Adults who were married in round 4 and became a widow/widower in round 5 were categorized as being widowed; those who were married in round 4 and were divorced in round 5 were categorized as being divorced.

#### Biological risk factors and chronic disease

BMI was calculated based on measured body weight and height and categorized into normal weight <25 kg/m^2^, overweight 25–29.9 kg/m^2^, and obesity ≥30 kg/m^2^ [32]. Multimorbidity was defined as having two or more chronic diseases [33] out of the following five self-reported diseases: diabetes, cancer, myocardial infarction, cerebrovascular accident, and chronic respiratory symptoms.

### Statistical analyses

Descriptive analyses were carried out for the total study population and for the physically, cognitively, psychologically and socially frail separately. For each frailty domain, we used logistic regression models to explore the factors associated with frailty. Odds ratios and 95% confidence intervals of two multivariable models are shown. The first model was adjusted for sociodemographic factors (model 1) and the second model was adjusted for all sociodemographic factors, lifestyle, life events, biological risk factors and chronic diseases (model 2). All analyses were carried out in SAS 9.3 (SAS Institute Inc., Cary, NC, USA).

## Results

In our population aged 40–81 years, 2.7% was physically frail, 6.3% was psychologically frail, 7.7% was cognitively frail, and 4.1% was socially frail (Table 2). Women were more frequently physically and psychologically frail (63.6% and 68.7%) than men (36.4% and 31.3% respectively), whereas men were more often cognitively frail than women (68.8% vs. 31.2%). The mean age of those with physical, cognitive and social frailty was higher compared to that of the total study population. Each of the domains of frailty showed a higher percentage of respondents with a low educational level compared to the total study population.

**Table 2:** Characteristics of the study population and persons being physically, psychologically, cognitively, and socially frail

	Study population (*N* = 4019)	Physically frail (*N* = 110; 2.7%)	Psychologically frail (*N* = 252; 6.3%)	Cognitively frail (*N* = 311; 7.7%)	Socially frail (*N* = 166; 4.1%)
Socio-demographic					
Women	2118 (52.7%)	70 (63.6%)	173 (68.7%)	97 (31.2%)	81 (48.8%)
Age, yr	59.9 (SD 9.6)	68.7 (SD 9.1)	59.3 (SD 9.9)	68.8 (SD 8.0)	63.0 (SD 10.4)
Level of education					
Low	1657 (41.2%)	76 (69.1%)	142 (56.3%)	154 (49.5%)	94 (56.6%)
Intermediate	1320 (32.9%)	14 (12.7%)	67 (26.6%)	85 (27.3%)	47 (28.3%)
High	1042 (25.9%)	20 (18.2%)	43 (17.1%)	72 (23.2%)	25 (15.1%)
Married	3211 (80.3%)	63 (57.3%)	151 (60.4%)	232 (75.3%)	108 (65.1%)
Living alone	555 (13.9%)	38 (34.6%)	63 (25.2%)	68 (22.2%)	43 (25.9%)
Paid job	2024 (50.5%)	7 (6.4%)	113 (44.8%)	52 (16.8%)	59 (35.8%)
Lifestyle					
Current smoking	682 (17.1%)	30 (27.8%)	77 (30.7%)	51 (16.6%)	34 (20.7%)
Healthy diet, score (0–7)	2.9 (SD 1.2)	2.6 (SD 1.1)	2.9 (SD 1.1)	2.8 (SD 1.2)	2.9 (SD 1.2)
Physically active	3112 (77.5%)	46 (41.8%)	170 (67.5%)	212 (68.4%)	108 (65.1%)
Sleep duration					
≤5 h	163 (4.1%)	12 (11.0%)	29 (11.5%)	15 (4.9%)	17 (10.3%)
6 h	703 (17.6%)	24 (22.0%)	71 (28.3%)	52 (16.8%)	36 (21.7%)
7 or 8 h	2880 (72.2%)	56 (51.4%)	127 (50.6%)	208 (67.3%)	95 (57.2%)
≥9 h	245 (6.1%)	17 (15.6%)	24 (9.6%)	34 (11.0%)	18 (10.8%)
Alcohol consumption					
Never	399 (10.0%)	28 (25.5%)	40 (15.9%)	39 (12.6%)	21 (12.7%)
Not anymore	124 (3.1%)	11 (10.0%)	16 (6.3%)	15 (4.8%)	9 (5.5%)
Low (<1 glass/wk)	849 (21.2%)	23 (20.9%)	61 (24.2%)	54 (17.4%)	39 (23.6%)
Frequent (≥1 glasses/wk)	2630 (65.7%)	48 (43.6%)	135 (53.6%)	202 (65.2%)	96 (58.2%)
Life events					
Widowed	74 (2.0%)	4 (4.0%)	12 (5.3%)	7 (2.5%)	4 (2.6%)
Divorced	66 (1.7%)	1 (1.0%)	10 (4.4%)	0 (0.0%)	7 (4.6%)
Biological risk factors and chronic disease					
BMI, kg/m^2^	26.8 (SD 4.2)	30.0 (SD 6.9)	27.4 (SD 5.0)	28.1 (SD 4.4)	27.1 (SD 4.7)
Multimorbidity^a^	320 (8.0%)	43 (39.1%)	40 (15.9%)	61 (19.6%)	26 (15.7%)
Frailty					
Physically frail	110 (2.7%)	-	34 (13.5%)	26 (8.4%)	19 (11.5%)
Psychologically frail	252 (6.3%)	34 (31.2%)	-	31 (10.0%)	52 (31.3%)
Cognitively frail	311 (7.7%)	26 (23.6%)	31 (12.3%)	-	20 (12.0%)
Socially frail	166 (4.1%)	19 (17.3%)	52 (20.6%)	20 (6.5%)	-

Frequencies (percentage) or means (SD) are presented

Note: *BMI* body mass index, *SD* standard deviation

^a^Multimorbidity was defined as having two or more conditions out of diabetes, cancer, myocardial infarction, cerebrovascular accident, and chronic respiratory symptoms

17.1% of the population was frail on one or more of the domains. Of the frail population, 81.5% was frail on one of the domains, 15.2% was frail on two domains, 2.9% was frail on three domains, and 0.4% was frail on all four domains. The greatest overlap was observed for physical and psychological frailty and for social and psychological frailty (one third of the physically and socially frail being also psychologically frail) (Table 2).

### Factors associated with physical, psychological, cognitive, and social frailty

An intermediate level of education, a paid job, a healthy diet, being physically active, and frequent alcohol consumption were associated with a lower risk of being *physically frail*, whereas, being 70 to 81 years, current smoking, a short sleep duration, and multimorbidity were associated with a higher risk of being physically frail (Table 3). Having a paid job showed the strongest negative association with being physically frail (Odds Ratio (OR) 0.16 (95% Confidence Interval (CI) 0.07–0.41)).

**Table 3:** Results of the logistic regression analyses on the associated factors with physical, cognitive, psychological, and social frailty

	Physically frail		Psychologically frail		Cognitively frail		Socially frail	
Variables	Model 1^d^ OR (95% CI)	Model 2^e^ OR (95% CI)	Model 1 OR (95% CI)	Model 2 OR (95% CI)	Model 1 OR (95% CI)	Model 2 OR (95% CI)	Model 1 OR (95% CI)	Model 2 OR (95% CI)
Socio-demographic								
Women	1.10 (0.71 1.68)		**1.67 (1.26 2.21)**	**1.90 (1.37 2.63)**	**0.29 (0.22 0.39)**	**0.26 (0.19 0.36)**	**0.69 (0.49 0.95)**	**0.59 (0.41 0.85)**
Age								
40–49 yr^a^	1	1	1		-	-	1	
50–59 yr^a^	1.61 (0.53 4.92)	1.93 (0.53 7.04)	1.02 (0.70 1.49)		1	1	1.10 (0.65 1.86)	
60–69 yr	1.23 (0.40 3.76)	1.79 (0.48 6.62)	0.70 (0.44 1.10)		1.35 (0.89 2.06)	1.28 (0.82 2.00)	0.97 (0.53 1.78)	
70–81 yr	**3.00 (1.00 9.07)**	**3.89 (1.05 14.42)**	**0.50 (0.29 0.86)**		**3.91 (2.50 6.12)**	**3.32 (2.06 5.35)**	1.46 (0.76 2.80)	
Level of education								
Low	1.58 (0.94 2.66)	0.88 (0.49 1.57)	**1.88 (1.31 2.71)**	**1.52 (1.03 2.24)**	**1.49 (1.08 2.06)**		**2.24 (1.42 3.54)**	**2.06 (1.27 3.34)**
Intermediate	0.60 (0.30 1.22)	**0.42 (0.21 0.95)**	1.20 (0.80 1.78)	0.93 (0.60 1.43)	1.15 (0.81 1.64)		1.56 (0.95 2.56)	1.31 (0.77 2.23)
High	1	1	1	1	1		1	1
Married	**0.36 (0.18 0.72)**		**0.30 (0.21 0.45)**	**0.34 (0.22 0.55)**	0.74 (0.42 1.31)		**0.48 (0.28 0.82)**	**0.47 (0.26 0.87)**
Living alone	0.80 (0.38 1.70)		0.82 (0.52 1.30)		1.13 (0.62 2.07)		1.04 (0.57 1.91)	
Paid job	**0.11 (0.04 0.26)**	**0.16 (0.07 0.41)**	**0.69 (0.49 0.98)**		**0.42 (0.28 0.64)**	**0.36 (0.23 0.56)**	0.66 (0.42 1.04)	
Lifestyle								
Current smoking	**2.55 (1.60 4.07)**	**3.18 (1.87 5.42)**	**1.93 (1.43 2.59)**	**1.92 (1.37 2.67)**	1.53 (1.08 2.17)		1.25 (0.84 1.86)	
Healthy diet	**0.79 (0.67 0.94)**	**0.78 (0.64 0.94)**	1.06 (0.95 1.18)		**0.87 (0.78 0.97)**	**0.88 (0.79 0.99)**	1.00 (0.88 1.14)	
Physically active	**0.22 (0.14 0.32)**	**0.32 (0.20 0.51)**	**0.62 (0.47 0.82)**	**0.71 (0.52 0.98)**	**0.50 (0.38 0.66)**	**0.60 (0.40 0.82)**	**0.58 (0.42 0.82)**	**0.60 (0.41 0.86)**
Sleep duration								
≤ 5 h	**2.97 (1.51 5.84)**	**2.44 (1.10 5.43)**	**4.14 (2.62 6.55)**	**4.25 (2.58 6.98)**	1.36 (0.75 2.46)		**2.89 (1.66 5.05)**	**3.11 (1.73 5.60)**
6 h	**1.84 (1.11 3.05)**	1.54 (0.86 2.75)	**2.36 (1.73 3.22)**	**2.39 (1.72 3.34)**	1.20 (0.85 1.69)		**1.55 (1.04 2.31)**	1.38 (0.89 2.14)
7 or 8 h	1	1	1	1	1		1	1
≥ 9 h	**2.36 (1.32 4.21)**	1.66 (0.86 3.22)	**2.42 (1.50 3.91)**	**2.11 (1.24 3.59)**	1.40 (0.90 2.15)		**1.93 (1.13 3.30)**	1.71 (0.96 3.05)
Alcohol consumption								
Never	1	1	1		1		1	
Not anymore	1.38 (0.62 3.06)	1.46 (0.59 3.60)	1.61 (0.84 3.06)		0.86 (0.43 1.73)		1.14 (0.48 2.68)	
Low (<1 glass/wk)	**0.54 (0.30 0.97)**	0.55 (0.27 1.12)	0.70 (0.46 1.08)		**0.60 (0.37 0.96)**		0.97 (0.56 1.70)	
Frequent (≥1 glasses/wk)	**0.40 (0.24 0.68)**	**0.53 (0.29 0.98)**	**0.65 (0.44 0.96)**		**0.60 (0.40 0.91)**		0.78 (0.47 1.31)	
Life events								
Widowed	0.51 (0.17 1.54)		1.48 (0.74 2.97)		0.60 (0.25 1.42)		0.51 (0.17 1.48)	
Divorced^b^	0.76 (0.10 5.95)		1.30 (0.62 2.74)		-		2.15 (0.89 5.17)	
Biological risk factors and chronic disease								
BMI								
Normal (<25 kg/m^2^)	1		1		1		1	
Overweight (25–30 kg/m^2^)	0.67 (0.39 1.14)		1.25 (0.92 1.71)		1.29 (0.94 1.76)		0.78 (0.54 1.12)	
Obese (≥30 kg/m^2^)	**2.12 (1.29 3.47)**		1.24 (0.86 1.80)		**1.80 (1.26 2.59)**		0.72 (0.46 1.14)	
Multimorbidity^c^	**4.47 (2.95 6.79)**	**3.81 (2.32 6.26)**	**2.22 (1.55 3.18)**	**1.83 (1.16 2.87)**	**1.71 (1.25 2.34)**		1.30 (0.84 2.02)	

Odds ratios and 95% confidence intervals are presented in this table. Bold values indicate significance (*p* < 0.05)

*Note: BMI* body mass index, *OR* odds ratio, 95% *CI* 95% confidence interval

^a^For cognitively frail: age categories are 45–59 yr., 60–69 yr., 70–81 yr., 45–59 yr. is the reference category

^b^For cognitively frail: no respondents were divorced

^c^Multimorbidity was defined as having two or more conditions out of diabetes, cancer, myocardial infarction, cerebrovascular accident, and chronic respiratory symptoms

^d^Model 1 are multivariate models adjusted for socio-demographic variables: sex, age, level of education, marital status, living situation, job status

^e^Model 2 are multivariate models adjusted for socio-demographic variables, lifestyle, life-events, biological risk factors, and chronic disease (all variables in the table). For model 2 only statistically significant odds ratios are presented to increase readability of the table

A higher risk of being *psychologically frail* was observed for the following factors: female sex, low educational level, current smoking, short and long sleep duration, and multimorbidity. Being married and being physically active were associated with a lower risk of being psychologically frail. A short sleep duration (≤5 h: 4.25 (95% CI 2.58–6.98); 6 h: 2.39 (95% CI 1.72 3.34)) and a long sleep duration (≥9 h: 2.11 (95% CI 1.24–3.59)) were consistently associated with psychological frailty.

Being 70 to 81 years (0.26 95% CI 0.19–0.36) was the only factor associated with a higher risk of being *cognitively frail*. Being female, a paid job, a healthy diet, and being physically active were associated with a lower risk of being cognitively frail. A low educational level and short sleep duration were associated with a higher risk of being *socially frail*. Being a female, being married, and being physically active were associated with a lower risk of being socially frail.

Overall, physical activity was consistently associated with a lower risk of being frail on all four domains. Short sleep duration was consistently associated with three out of the four frailty domains. Living alone, life events, and overweight or obesity were not associated with any of the domains of frailty in the multivariable model.

### Frailty on one or more domains

A higher age, a low level of education, current smoking, short and long sleep duration, and multimorbidity were associated with a higher risk to be frail on one or more of the four domains (Table in Additional file 2). Being a female, being married, having a paid job, having a healthy diet, being physically active, and consuming alcohol (≥1 glasses/wk) were associated with a lower risk to be frail on one or more domains.

## Discussion

This study suggests that each of the different frailty domains all had a specific combination of associated factors. Most socio-demographic factors and lifestyle were associated with being frail on each of the domains. Being physically active was consistently associated with a lower risk of being frail on each of the four domains. A short or long sleep duration was associated with a higher risk of being physically, psychologically, and socially frail. Other factors associated with one or more domains of frailty were female sex, high age, a low educational level, being married, a paid job, current smoking, a healthy diet, and multimorbidity.

Drawing on the integral conceptual model of frailty, we observed a prevalence of 17.1% among men and women of 40 to 81 years who lived independently. The proportion of frail persons in a population is dependent on the definition of frailty used [34] and on characteristics of the study population. The prevalence of frailty that we observed is relatively low compared to recent other studies based on the integral conceptual model of frailty [21, 22, 35], which could be explained by the large age range of our population and the absence of persons over 81 years of age. The majority of studies directed to frailty have focused solely on people over the age of 65, despite emerging evidence suggesting that frailty begins much earlier than that [36, 37]. Our findings show that frailty may already exist at a relative young age and therefore extend the findings of previous studies.

The relationship between socio-demographic factors and physical frailty has been described in the literature [13, 38]. Like age, sex also contributed differently to each of the frailty domains: being female was associated with a higher risk of being psychologically frail and a lower risk of being cognitively and socially frail. A recent review showed socioeconomic status to be inversely associated with physical frailty [13]. Our findings support a higher risk of being psychologically and socially frail for people with low education. Briefly, socio-demographic factors are important for frailty but their impact varied for each of the domains of frailty.

Lifestyle factors in relation to multiple domains of frailty have not (yet) been studied extensively. An unhealthy lifestyle was previously found to be associated with a higher risk of being physically and socially frail [39–41], and psychologically frail (including cognitive frailty) [39, 40]. However, in these studies lifestyle was assessed by a single item in a self-report questionnaire. Such assessment of lifestyle precludes unambiguous interpretation, because it remains unclear which lifestyle factors participants had in mind when answering the question and what aspect they considered to be unhealthy [40]. Our findings provide novel insight into the specific lifestyle factors (physical activity, smoking, diet, alcohol consumption, sleep) associated with the different domains of frailty. Physical activity was significantly associated with all domains of frailty in our study. A previous study of Strawbridge also considered a broad range of risk factors including lifestyle, in relation to a multidimensional definition of frailty [42]. Being physically inactive, either at one instant or at several measurements over a period of 29 years, was associated with a higher risk of being frail. Other studies confirmed the associations of physical activity with physical frailty and cognitive decline [43, 44], as far as we know no studies included the domains of psychological and social frailty. Our results are in line with earlier studies showing that current smoking was associated with a higher risk of being physically frail [45], and a healthy diet was associated with a lower risk of being physically and cognitively frail [46, 47]. A new insight based on our findings is that short sleep duration was associated with a higher risk to be physically, psychologically and socially frail, and long sleep duration was associated with a higher risk to be psychologically frail. Sleep deprivation contributes to a number of molecular, immune and neural changes that play a role in the development of health problems [48]. Previously, sleep quality and sleep disturbances, but not sleep duration, were reported to be associated with physical frailty [49]. More detailed studies are needed to understand the relation between sleep and each of the frailty domains [50].

In addition to sociodemographic factors and lifestyle, we studied life events, multimorbidity and overweight. Life events were not associated with any of the frailty domains in our study. Other studies reported life events to be associated with a higher risk of being psychologically frail [39–41]. Overweight and obesity were not associated with frailty in our multivariate models, which is comparable to the findings of Strawbridge [42]. Some studies that adjusted for socio-demographic variables and smoking, but not for other lifestyle factors, did report an association between obesity and (physical) frailty [51, 52]. These inconsistent results regarding the relationship between life events and overweight with frailty can be explained by methodological differences such as the definition of life events. Finally, multimorbidity was associated with a higher risk to be physically and psychologically frail, which is in line with other studies [39–42]. Fried illustrated that frailty is distinct but overlapping with comorbidity, with almost 70% of the frail persons also having two or more diseases [8].

In general, there is no consensus about a definition of frailty [34, 53] as shown by the different approaches described in the literature. Besides the well-known frailty phenotype approach [1], the frailty index is another dominant approach in frailty research [54]. The frailty index involves the accumulation of diseases, symptoms, signs, disabilities or any deficiency in health with age [55]. Although different domains of human functioning are incorporated in the frailty index, it differs from our approach to frailty because it considers frailty as much broader than functioning alone. The incorporation of social functioning in the concept of frailty is an area of discussion. During the development of the integral conceptual model of frailty a group of frailty experts agreed upon including social functioning in this model [19]. The social domain cannot be left out because it is relevant to an integrated view of human beings [3, 56], the relationship with adverse outcomes is demonstrated [57, 58], and ‘social relationships’ and ‘social support’ are viewed as determinants of frailty [42, 59, 60]. Two other reasons to consider social functioning as part of a multidimensional definition of frailty are (1) social functioning is regarded as separate health domain in the Comprehensive Geriatric Assessment and therefore viewed as relevant in clinical practice [7], and (2) in a qualitative study the majority of interviewed elderly persons reported to consider reduced social functioning as an important component of frailty [61].

The Doetinchem Cohort Study is a unique cohort for studying frailty because of the relatively wide age range of the participants and the ability to define multiple frailty domains due to the wide array of collected variables. The original TFI scale is based on self-report, but we were able to combine self-reported and objectively measured variables to define frailty. Since the population has a wide age range and includes middle-aged adults, we used similar or more stringent cut-off points than applied earlier by van Campen [22]. A sensitivity analysis for cognitive frailty with a lower cut-off (<7.5%) confirmed the findings of cognitive frailty defined by 10th percentile, except for alcohol consumption, obesity and multimorbidity. Since data required to define social frailty (as well as some of the indicators for physical and psychological frailty) were measured for the first time in the most recent completed round of the Doetinchem Cohort Study, longitudinal analyses were not possible. As such, all analyses were cross-sectional and causal inferences cannot be made. To illustrate this, we take the example of having a paid job. We found that having a paid job was strongly associated with a lower risk to be physically frail. This may suggest a protective effect of having a paid job for being physically frail. However, it may also imply that frail persons have stopped working because of limitations in work functioning. Future studies are needed to address the prospective association between a various range of factors and the development of physical, psychological, cognitive, and social frailty. Another limitation is the measurement of life events. Other studies have shown that especially death of a loved one was associated with frailty [39]. The questionnaire used for the Doetinchem Cohort study did not assess death of a loved one. We therefore decided to use the variable ‘being widowed’ as a proxy. We should however, acknowledge that the life events being widowed and death of a loved one may partly overlap but are not the same. This may explain the lack of association with psychological frailty. Although the response rates for the examination rounds varies between 75 and 80%, we cannot exclude selective response that may have caused an underrepresentation of severely frail participants, in particular of physically and cognitively frail individuals. To minimize a healthy cohort effect, home visits were offered if participants were not able to get to the municipal health services where the examinations were carried out. This way, frail participants could still participate in the study. However, due to potential selective response the observed associations may be underestimated.

## Conclusions

The present study suggests that frailty, in particular psychological frailty, may already be present at a relatively young age. Sociodemographic factors, lifestyle and multimorbidity contributed differently to each of the frailty domains. This highlights the relevance of a multidimensional approach to frailty as operationalized in the integral conceptual model of frailty. Understanding which groups of older adults are at risk of being frail on each domain may help to prevent frailty and to identify frail individuals in an early stage. Identification of frail individuals is an important step to preclude the development of undesirable outcomes, to provide adequate healthcare and support, and to effectively prevent and delay the development of frailty by health professionals. Since we know little about factors associated with the psychological, cognitive and social domains of frailty, our findings add to available literature and are relevant for clinical practice [34]. We found that lower educated adults were at higher risk of being frail, and therefore preventive strategies should be directed at this group. Furthermore, our findings suggest that lifestyle factors, specifically physical activity and sleep, are associated with the presence of frailty. As lifestyle is potentially modifiable, interventions directed to improve lifestyle may provide new opportunities for the prevention of frailty in the future. But first longitudinal research should be conducted to better examine how lifestyle affects the development of frailty and its dynamic course.

## Additional files

Additional file 1: Extensive description of the four domains of frailty. (DOCX 17 kb)

Additional file 2: Results of the logistic regression analyses on the associated factors with frailty on any of the domains. (DOCX 19 kb)

## Abbreviations

BMI: Body Mass Index
TFI: Tilburg frailty indicator
