# Supplementary material for: A four-domain approach of frailty explored in the Doetinchem Cohort Study
Source: BMC Geriatr. 2017 Aug 30;17:196. doi: 10.1186/s12877-017-0595-0 (PMC5577839; doi:10.1186/s12877-017-0595-0)
Supplement: Supplementary file 1 — Extensive description of the four domains of frailty. (DOCX 17 kb) [file 12877_2017_595_MOESM1_ESM.docx]

**Additional file 1**

**Methods**

Physical frailty

Participants were considered physically frail when they fulfilled the specified requirements for ≥4 of the following 8 criteria: exhaustion, unintentional weight loss, low handgrip strength, perceived health, walking, balance, poor hearing, and poor vision (Table 1)[21]. Unintentional weight loss was defined as weight loss >5% between round 4 and 5 and the participant reporting not to be on a diet. Presence of exhaustion was assessed using the following two statements of the Center for Epidemiologic Studies Depression scale (CES-D)[23]: “I felt that everything I did was an effort” and “I could not get going”[23]. Participants answering a ‘moderate amount of the time (3-4 days)’, or ‘most of the time’ to either of these questions were considered to be exhausted. Handgrip strength was measured using a dynamometer (Jamar, Sammons Preston Rolyan). Cut-off points for handgrip strength in kg were stratified by sex and BMI. Cut-off points for men were ≤29.0 kg for BMI ≤24.0, ≤30.0 kg for BMI 24.1-26.0, ≤30 kg for BMI 26.1-28.0, and ≤32.0 kg for BMI >28.0. Cut-off points for women were ≤17.0 kg for BMI ≤23.0, ≤17.3 kg for BMI 23.1-26.0, ≤18.0 kg for BMI 26.1-29.0, and ≤21.0 kg for BMI >29.0. The cut-off points used for exhaustion and handgrip strength were based on Fried[1]. Limitations in walking due to health were assessed with a question regarding 100 meter walking. Poor perceived self-reported health were those with a self-reported health of less than good, on a scale with the following response categories: poor, fair, good, very good, excellent. The question was taken from the 36-Item Short-Form Health Survey (SF-36)[24, 62]. The Tandem Stand Balance Test was used to test balance in participants ≥60 years old. Failing or refusing to do the test, or not being able to keep balance >10 seconds, was considered a positive score for this criterion (being <60 years counted as a negative score). Hearing impairment (present or absent) was also assessed by self-report, with questions on hearing in general, and experienced difficulties in group conversations. Vision impairment (present or absent) was assessed in a similar manner.

Cognitive frailty

Participants were considered cognitively frail when scoring <10^th^ percentile on global cognitive functioning (Table 1). Global cognitive functioning was assessed with a neuropsychological test battery. Memory function, information processing speed and cognitive flexibility were tested using 15-words Verbal Learning Test, the Stroop Colour–Word Test, the Word Fluency Test and the Letter Digit Substitution Test. Nooyens et al. describe the cognitive tests in more detail[63]. The cognitive tests were performed among participants ≥45 years old. Cognitive scores were adjusted for level of education and number of tests performed during follow-up.

Psychological frailty

Psychological frailty was defined as fulfilling both criteria for depression and criteria for general mental health (Table 1). Depressive symptoms were assessed with the CES-D. Those with a CES-D score of ≥16 (out of range of 0-60) were defined to have a high risk of depression. Mental health status was measured with the Mental Health Inventory 5 (MHI-5)[24]. Scores of five questions on a six-point scale were transformed into a total score ranging from 0 to 100 and a cut-off point of ≤60 was used to indicate poor mental health status[64]. Coping was no criterion for the psychological domain because it was not available.

Social frailty

Social frailty was defined as meeting ≥2 of 3 criteria using the Loneliness scale, Social Support List-12 and a questionnaire about social participation from the Dutch Municipal Health Services Elderly Monitor (Table 1). A cut-off point of ≥9 on the Loneliness scale (11 items with a score 0 or 1) was used to indicate loneliness[25, 65]. A score of <24 (out of 48) on the 12-item Social Support List was considered to indicate low social support[26]. Finally, participants in the lowest decile on The Dutch Municipal Health Services Elderly Monitor[27], a questionnaire regarding social participation used in the Dutch public health care for elderly people (15 items with a maximum score of 65), were considered to have limited social participation. Living alone was not included in the definition of the social domain of frailty because we studied living alone as a determinant of all frailty domains.
