# Supplementary material for: A four-domain approach of frailty explored in the Doetinchem Cohort Study
Source: BMC Geriatr. 2017 Aug 30;17:196. doi: 10.1186/s12877-017-0595-0 (PMC5577839; doi:10.1186/s12877-017-0595-0)
Supplement: Supplementary file 2 — Results of the logistic regression analyses on the associated factors with frailty on any of the domains. (DOCX 19 kb) [file 12877_2017_595_MOESM2_ESM.docx]

**Table.** Results of the logistic regression analyses on the associated factors with frailty on any of the domains.

|  | **Frail on one or more domains** | |
| --- | --- | --- |
| **Variables** | Model 1^2^  OR (95% CI) | Model 2^3^  OR (95% CI) |
| **Socio-demographic** |  |  |
| Women | **0.67 (0.56 0.81)** | **0.62 (0.51 0.77)** |
| Age |  |  |
| 40-49 yr | 1 | 1 |
| 50-59 yr | **1.49 (1.09 2.05)** | **1.70 (1.20 2.41)** |
| 60-69 yr | 1.39 (0.98 1.98) | **1.74 (1.17 2.58)** |
| 70-81 yr | **2.99 (2.05 4.35)** | **3.70 (2.42 5.66)** |
| Level of education |  |  |
| Low | **1.62 (1.29 2.04)** | **1.29 (1.01 1.65)** |
| Intermediate | 1.20 (0.94 1.54) | 1.03 (0.79 1.34) |
| High | 1 | 1 |
| Married | **0.42 (0.31 0.57)** | **0.41 (0.28 0.58)** |
| Living alone | 0.78 (0.55 1.12) | 0.71 (0.47 1.06) |
| Paid job | **0.50 (0.39 0.65)** | **0.58 (0.44 0.77)** |
|  |  |  |
| **Lifestyle** |  |  |
| Current smoking | **1.69 (1.36 2.10)** | **1.60 (1.26 2.04)** |
| Healthy diet | **0.93 (0.86 1.00)** | **0.93 (0.86 1.00)** |
| Physically active | **0.52 (0.43 0.63)** | **0.62 (0.50 0.76)** |
| Sleep duration |  |  |
| ≤5 hr | **2.56 (1.77 3.69)** | **2.30 (1.55 3.43)** |
| 6 hr | **1.68 (1.35 2.10)** | **1.53 (1.20 1.94)** |
| 7 or 8 hr | 1 | 1 |
| ≥9 hr | **2.02 (1.49 2.76)** | **1.76 (1.26 2.46)** |
| Alcohol consumption |  |  |
| Never | 1 | 1 |
| Not anymore | 1.07 (0.66 1.73) | 1.20 (0.71 2.02) |
| Low (<1 glass/wk) | 0.74 (0.54 1.00) | 0.86 (0.61 1.21) |
| Frequent (≥1 glasses/wk) | **0.59 (0.45 0.78)** | **0.71 (0.52 0.97)** |
|  |  |  |
| **Life events** |  |  |
| Widowed | 0.84 (0.48 1.47) | 1.12 (0.63 2.00) |
| Divorced | 1.01 (0.51 1.98) | 1.08 (0.54 2.17) |
|  |  |  |
| **Biological risk factors and chronic disease** |  |  |
| BMI |  |  |
| Normal (<25 kg/m^2^) | 1 | 1 |
| Overweight (25-30 kg/m^2^) | 1.09 (0.89 1.34) | 1.01 (0.81 1.26) |
| Obese (≥30 kg/m^2^) | **1.35 (1.06 1.71)** | 1.01 (0.77 1.33) |
| Multimorbidity^1^ | **2.38 (1.84 3.09)** | **1.89 (1.42 2.52)** |

Odds ratios and 95% confidence intervals are presented in this table.

^1^ Multimorbidity was defined as having two or more out of diabetes, cancer, myocardial infarction, cerebrovascular accident, and chronic respiratory symptoms.

^2^ Model 1 are multivariate models adjusted for socio-demographic variables: sex, age, level of education, marital status, living situation, job status.

^3^ Model 2 are multivariate models adjusted for socio-demographic variables, lifestyle, life-events, biological risk factors, and chronic disease (all variables in the table).

*Note:* BMI=body mass index, OR= odds ratio, 95% CI= 95% confidence interval
